# Perinatal Psychotherapy Use and Costs Before and After Federally Mandated Health Insurance Coverage

**DOI:** 10.1001/jamanetworkopen.2024.26802

**Published:** 2024-08-09

**Authors:** Kara Zivin, Xiaosong Zhang, Anca Tilea, Stephanie V. Hall, Lindsay K. Admon, Ashlee J. Vance, Vanessa K. Dalton

**Affiliations:** 1 Center for Clinical Management Research, Veterans Affairs Ann Arbor Healthcare System, Ann Arbor, Michigan; 2 Department of Psychiatry, Michigan Medicine, Ann Arbor; 3 Department of Obstetrics and Gynecology, Michigan Medicine, Ann Arbor; 4 Department of Learning Health Sciences, Michigan Medicine, Ann Arbor; 5 Center for Health Policy and Health Services Research, Henry Ford Health, Detroit, Michigan

## Abstract

**Importance:**

Insurance coverage affects health care access for many delivering women diagnosed with perinatal mood and anxiety disorders (PMADs). The Mental Health Parity and Addiction Equity Act (MHPAEA; passed in 2008) and the Patient Protection and Affordable Care Act (ACA; passed in 2010) aimed to improve health care access.

**Objective:**

To assess associations between MHPAEA and ACA implementation and psychotherapy use and costs among delivering women overall and with PMADs.

**Design, Setting, and Participants:**

This cross-sectional study conducted interrupted time series analyses of private insurance data from January 1, 2007, to December 31, 2019, for delivering women aged 15 to 44 years, including those with PMADs, to assess changes in psychotherapy visits in the year before and the year after delivery. It estimated changes in any psychotherapy use and per-visit out-of-pocket costs (OOPCs) for psychotherapy associated with MHPAEA (January 2010) and ACA (January 2014) implementation. Data analyses were performed from August 2022 to May 2023.

**Exposures:**

Implementation of the MHPAEA and ACA.

**Main Outcomes and Measures:**

Any psychotherapy use and per-visit OOPCs for psychotherapy standardized to 2019 dollars.

**Results:**

The study included 837 316 overall deliveries among 716 052 women (mean [SD] age, 31.2 [5.4] years; 7.6% Asian, 8.8% Black, 12.8% Hispanic, 64.1% White, and 6.7% unknown race and ethnicity). In the overall cohort, a nonsignificant step change was found in the delivering women who received psychotherapy after MHPAEA implementation of 0.09% (95% CI, −0.04% to 0.21%; *P* = .16) and a nonsignificant slope change of delivering women who received psychotherapy of 0.00% per month (95% CI, −0.02% to 0.01%; *P* = .69). A nonsignificant step change was found in delivering individuals who received psychotherapy after ACA implementation of 0.11% (95% CI, −0.01% to 0.22%; *P* = .07) and a significantly increased slope change of delivering individuals who received psychotherapy of 0.03% per month (95% CI, 0.00% to 0.05%; *P* = .02). Among those with PMADs, the MHPAEA was associated with an immediate increase (0.72%; 95% CI, 0.26% to 1.18%; *P* = .002) then sustained decrease (−0.05%; −0.09% to −0.02%; *P* = .001) in psychotherapy receipt; the ACA was associated with immediate (0.77%; 95% CI, 0.26% to 1.27%; *P* = .003) and sustained (0.07%; 95% CI, 0.02% to 0.12%; *P* = .005) monthly increases. In both populations, per-visit monthly psychotherapy OOPCs decreased (−$0.15; 95% CI, −$0.24 to −$0.07; *P* < .001 for overall and −$0.22; −$0.32 to −$0.12; *P* < .001 for the PMAD population) after MHPAEA passage with an immediate increase ($3.14 [95% CI, $1.56-$4.73]; *P* < .001 and $2.54 [95% CI, $0.54-$4.54]; *P* = .01) and steady monthly increase ($0.07 [95% CI, $0.02-$0.12]; *P* = .006 and $0.10 [95% CI, $0.03-$0.17]; *P* = .004) after ACA passage.

**Conclusions and Relevance:**

This study found complementary and complex associations between passage of the MHPAEA and ACA and access to psychotherapy among delivering individuals. These findings indicate the value of continuing efforts to improve access to mental health treatment for this population.

## Key Points

**Question:**

Did psychotherapy use and out-of-pocket costs for delivering women change after passage of the Mental Health Parity and Addiction Equity Act (MHPAEA) and the Patient Protection and Affordable Care Act (ACA)?

**Findings:**

In this cross-sectional study of 837 316 overall deliveries among 716 052 women, interrupted time series analyses using US private insurance data showed that psychotherapy out-of-pocket costs exhibited seasonal variation that decreased after passage of the MHPAEA with an immediate, steady increase after passage of the ACA. Among those with perinatal mood and anxiety disorders, the MHPAEA was associated with an immediate decrease then sustained increase in psychotherapy; the ACA was associated with immediate, sustained increases.

**Meaning:**

These findings indicate that passage of the MHPAEA and ACA were associated with mixed effects on psychotherapy access among delivering individuals.

## Teaser

This cross-sectional study examines the association of changes in federal health legislation with mental health treatment and spending among delivering women in the US.

## Introduction

Perinatal mood and anxiety disorders (PMADs), including depression and/or anxiety diagnoses in the year before or after delivery, represent common pregnancy complications. These disorders affect up to 1 in 5 women and cost more than $15 billion per year in the US.^1,2,3,4^ Perinatal mood and anxiety disorders are associated with adverse gestational outcomes, such as preterm birth and low birth weight, are linked to maternal suicide, and represent a leading cause of postpartum maternal mortality.^5,6^ Perinatal mood and anxiety disorders remain among the leading causes of US pregnancy-related deaths,^7^ which have steadily risen.^8^ Perinatal mood and anxiety disorders can negatively affect mothers, infants, and families beyond the perinatal period, with lasting clinical and economic effects.^9^ Treatment for PMADs, including first-line treatment for mild to moderate illness, such as psychotherapy,^10^ can improve maternal and neonatal health outcomes,^11,12,13,14,15^ yet treatment remains rare.^16^

One potential reason for underuse of treatment for mental health (MH) disorders during the perinatal period is insufficient insurance coverage. Costs represent a leading barrier to MH care, contributing to unmet needs.^17^ Improving access to perinatal care by lowering costs could improve MH coverage obstacles, increase treatment for PMADs, and lead to positive effects on patients, payers, and health care spending.

Federal health legislation, including the Mental Health Parity and Addiction Equity Act of 2008 (MHPAEA; implemented in 2010) and the Patient Protection and Affordable Care Act of 2010 (ACA; implemented 2014), provided one of the largest expansions of MH coverage in a generation. Together, the MHPAEA and ACA increased coverage and extended federal parity protections to at least 60 million individuals in the US.^18^ Before implementation of the MHPAEA, most health plans, including commercial and employer-based plans, provided limited benefits for MH services. The MHPAEA requires plans to cover MH services and prohibits plans from providing less generous coverage benefits relative to medical-surgical care.

Unlike the MHPAEA, the ACA required MH coverage as 1 of 10 essential health benefits, and the ACA closed loopholes from the MHPAEA for which plans did not have to comply with coverage at parity, including extending parity protections to most individual and small-group plans.^18,19^ The ACA included features that may have indirectly improved access to MH coverage, such as Medicaid expansion, coverage on a parent’s plan until 26 years of age, prohibition on lifetime limits, and ending preexisting condition exclusions. Our group found that the ACA may have led to improved detection of PMADs in Michigan Medicaid.^20^ Others have found maternal MH improvements after ACA’s Medicaid expansion.^21^

The effects of ACA provisions would likely increase over time as fewer individuals remain in grandfathered health plans not required to comply with essential health benefits of the ACA. Whereas 72% of firms offering health benefits had at least 1 grandfathered plan in 2011, that percentage decreased to 16% by 2020.^22,23^ Given that at least 50% of US pregnant women have private health insurance,^24^ improvements in coverage due to MH policy changes could have broad implications for perinatal MH service access. Our group has found that MHPAEA and ACA may have lowered severe maternal morbidity, particularly among those with PMADs.^25^

In this context, we conducted the Maternal Behavioral Health Policy Evaluation (MAPLE) study using monthly analyses among all delivering women and those with diagnosed PMADs to examine the association of changes in federal health legislation with MH treatment and spending. This study takes advantage of a natural experiment using a large national sample of women enrolled in employer-based insurance. We hypothesized that the MHPAEA would have some cost and utilization benefits, with greater improvements after implementation of the ACA.

## Methods

### Study Design

We conducted a serial, cross-sectional analysis of women with inpatient deliveries resulting in live birth from 2007 to 2019 using Optum’s deidentified Clinformatics Data Mart Database (CDM). The CDM is a statistically deidentified database of administrative medical claims for a national, commercially insured population. We created a patient-level cohort of women with continuous enrollment in a single health plan for 12 months before and 12 months after delivery and with a maternal age of 15 to 44 years.^26^ We chose this time window given uncertainty about pregnancy length and timing surrounding conception,^27^ noting that 12 months represents a common timeframe in antenatal research.^28^ The largest perinatal surveillance system, the Pregnancy Risk Assessment Monitoring System, gathers data on health behaviors, utilization, and diagnosis in the 12 months before delivery.^29^ All cohort identification, diagnosis, and procedure codes appear in eTable 1 in Supplement 1. The study site’s institutional review board approved this deidentified data analysis. The study site's institutional review board approved the study with a waiver of informed consent because this is a secondary analysis of existing data. We followed the Strengthening the Reporting of Observational Studies in Epidemiology (STROBE) reporting guideline for cross-sectional studies.^30^

We included hospital deliveries using standardized *International Classification of Diseases, Ninth Revision, Clinical Modification* (*ICD-9-CM*) and *International Statistical Classification of Diseases, Tenth Revision, Clinical Modification* (*ICD-10-CM*) diagnosis and procedure codes, diagnosis-related group codes, and *Current Procedural Terminology* codes.^31,32,33^ We identified PMAD diagnoses in any diagnostic field (ie, not just principal diagnosis) at any point during the 2-year observation period surrounding delivery using Healthcare Cost and Utilization Project algorithms.^34,35^

To create our denominator, for every month in our analytical period, we determined whether a woman had a delivery anytime within the 12 months before and 12 months after the delivery month under consideration. We repeated this assessment for every month between January 1, 2006, and December 31, 2020, reflecting deliveries between January 1, 2007, and December 31, 2019. To create our numerator, we looked for evidence of our outcomes of interest in each month among individuals in the denominator for that month. After generating our denominators and numerators, we summarized our findings by month. For example, the numerator indicates individuals with any psychotherapy visit in January 2012; we would consider January 2010 the index month (month zero). The corresponding denominator includes individuals with a delivery during 24 months surrounding the index month (ie, January 1, 2011, to January 31, 2013) (eFigure 1 in Supplement 1).

### Study Outcomes and Variables

We generated 2 primary and 3 secondary outcomes. Primary outcomes included (1) percentage of women who received any psychotherapy and (2) per-visit out-of-pocket cost (OOPC) for psychotherapy. We focused on specialized MH treatment (psychotherapy), attempting to isolate effects of policy changes on MH treatment utilization (as distinct from obstetric, primary care, or other health services). We defined the first primary outcome (percentage of women receiving psychotherapy) by identifying psychotherapy visits using *Current Procedural Terminology* and Healthcare Common Procedure Coding System codes.^36^ We defined the second primary outcome, per-visit OOPC for psychotherapy, by summing the copayment, coinsurance, and deductible for psychotherapy visits with 2019 inflation–adjusted dollars using the Consumer Price Index.^37^ Secondary outcomes used in descriptive analyses included (1) total number of psychotherapy visits per month; (2) number of visits among women with 1 or more psychotherapy visits; (3) per-visit standard cost for psychotherapy, where standard cost reflects what the plan pays for the service (total amount paid for a service includes OOPC plus standard cost); (4) per-visit OOPCs and standard costs of psychotherapy visits per month per visit; and (5) ratio between OOPCs and standard costs of psychotherapy visits per month.

We used an interrupted time series analysis with autoregressive integrated moving average (ARIMA) models to evaluate changes in monthly outcomes, controlling for correlation with prior months (autoregression) and seasonality.^38,39^ We created intervention indicators for MHPAEA implementation in January 2010 and ACA implementation in January 2014.

We included MHPAEA and ACA implementation in models using 4 independent variables: (1) a dichotomous indicator (known as a step, level, or immediate change) for before and after MHPAEA implementation, which represented the overall change in outcomes from pre-MHPAEA to post-MHPAEA implementation; (2) a count indicator (known as a ramp, slope, or sustained change) for each month after MHPAEA implementation, which represented how the post-MHPAEA period month-to-month change differed from the pre-MHPAEA period; (3) a dichotomous indicator (step) for before and after ACA implementation; and (4) a count indicator (ramp) for months after ACA implementation, which represents the overall pre- to post-ACA change in outcomes and the relative difference in the month-to-month change in outcomes in the post-ACA period compared with pre-ACA.

The 4 independent variables covered 3 study periods. Period 1 represented the pre-MHPAEA and pre-ACA implementation period from January 1, 2007, through December 31, 2009. Period 2 represented the post-MHPAEA and pre-ACA period from January 1, 2010, through December 31, 2013. Period 3 represented the post-MHPAEA and post-ACA period from January 1, 2014, through December 31, 2019.

### Demographic and Clinical Characteristics

We assessed several demographic and clinical characteristics in our cohort, obtained through the CDM. Demographic characteristics include age (categorized as 15-24, 25-39, and 40-44 years to align with Centers for Disease Control and Prevention maternal mortality rate statistics for 2020),^40^ race and ethnicity (Asian, Black, Hispanic, White, or unknown), region (Midwest, Northeast, South, West, or unknown), and insurance plan type (exclusive provider organization, health maintenance organization, indemnity, preferred provider organization, point of service, or other). We included race and ethnicity to describe differences in psychotherapy use and costs by racial and ethnic group. Clinical characteristics include mode of delivery (cesarean or vaginal), diagnosis of suicidality, bipolar disorder, schizophrenia, substance use disorder, and medical comorbidity using the Bateman comorbidity index (score of 0 vs ≥1).^41^

### Statistical Analysis

We conducted analyses between August 2022 and May 2023. We evaluated ARIMA models among all deliveries and specifically among deliveries to women with PMADs. We made final ARIMA model selections based on inspection of model residuals and fit statistics.^42,43^ We included autoregressive, seasonal autoregressive, moving average, and seasonal moving average parameters to account for the potential seasonal nature of psychotherapy use and OOPCs associated with health care use and health plan coverage. We created the study cohort and variables using SAS software, version 9.4 (SAS Institute Inc) and conducted analyses using R software, version 4.2.0 (R Foundation for Statistical Computing) using the forecast package (version 8.18)^44^ and astsa (applied statistical time series analysis) package (version 1.16).^45^ A 2-sided *P* < .05 was considered statistically significant.

## Results

We identified 837 316 deliveries among 716 052 women (mean [SD] age, 31.2 [5.4] years; 7.6% Asian, 8.8% Black, 12.8% Hispanic, 64.1% White, and 6.7% unknown race and ethnicity) during the study period, including 152 340 deliveries among 141 170 women with PMADs; 19.7% of the women experienced PMADs. Characteristics of the full study sample remained relatively stable over time (Table 1). The cohort in 2007 included 9.7% aged 15 to 24 years, 84.9% aged 25 to 39 years, and 5.4% aged 40 to 44 years. In 2019, the age distribution stayed similar to 2007. Race and ethnicity groups remained comparable in 2007 and 2019 as follows: 6.5% and 6.8% Asian, 8.3% and 7.6% Black, 11.9% and 12.6% Hispanic, 60.4% and 58.3% White, and 13.0% and 14.8% unknown in 2007 and 2019, respectively. Use of point-of-service insurance plans increased from 62.6% in 2007 to 73.4% in 2019, with corresponding decreases in other plan types. Physical and MH comorbidities increased over time. The proportion of women with a Bateman comorbidity index score greater than 0 nearly doubled from 15.2% in 2007 to 29.3% in 2019. Diagnoses of PMADs increased from 14.3% in 2007 to 25.2% in 2019 (eTable 2 in Supplement 1).

**Table 1.** Unadjusted Sociodemographic and Clinical Characteristics Associated With Deliveries Among Privately Insured Delivering Women, 2007-2019

Characteristic	No. (%) of women			
	2007 (n = 60 084)	2010 (MHPAEA) (n = 61 618)	2014 (ACA) (n = 53 184)	2019 (n = 52 549)
Age group, y				
15-24	5825 (9.7)	5914 (9.6)	6439 (12.1)	5049 (9.6)
25-39	50 994 (84.9)	51 776 (84.1)	43 880 (82.5)	44 369 (84.4)
40-44	3213 (5.4)	3883 (6.3)	2847 (5.4)	3124 (6.0)
Race and ethnicity				
Asian	3924 (6.5)	4364 (7.1)	4585 (8.6)	3581 (6.8)
Black	4976 (8.3)	6125 (9.9)	4460 (8.4)	3982 (7.6)
Hispanic	7123 (11.9)	7640 (12.4)	7185 (13.5)	6597 (12.6)
White	36 260 (60.4)	38 934 (63.2)	35 411 (66.6)	30 621 (58.3)
Unknown	7801 (13.0)	4555 (7.4)	1543 (2.9)	7768 (14.8)
Region				
Midwest	14 831 (24.7)	14 335 (23.3)	14 672 (27.6)	14 562 (27.7)
Northeast	6110 (10.2)	6349 (10.3)	5649 (10.6)	5460 (10.4)
South	26 990 (44.9)	28 858 (46.8)	21 131 (39.7)	21 329 (40.6)
West	12 098 (20.1)	12 034 (19.5)	11 655 (21.9)	11 053 (21.0)
Unknown	55 (0.1)	42 (0.1)	77 (0.1)	145 (0.3)
Insurance plan type				
EPO	10 173 (16.9)	10 500 (17.0)	6029 (11.3)	6904 (13.1)
HMO	9454 (15.7)	7212 (11.7)	5018 (9.4)	5686 (10.8)
PPO, indemnity, or other insurance plan	2826 (4.7)	1351 (2.2)	1088 (2.0)	1364 (2.6)
POS	37 631 (62.6)	42 555 (69.1)	41 049 (77.2)	38 595 (73.4)
Clinical characteristics				
Mode of delivery: cesarean	21 713 (36.1)	23 532 (38.2)	18 451 (34.7)	17 445 (33.2)
Bateman comorbidity index score >0	9115 (15.2)	9605 (15.6)	7981 (15.0)	15 395 (29.3)
Anxiety	4811 (8.1)	5931 (9.6)	6612 (12.4)	11 294 (21.5)
Depression	5786 (9.8)	6377 (10.4)	5560 (10.5)	6689 (12.8)
Suicidality	112 (0.2)	167 (0.3)	229 (0.4)	413 (0.8)
Bipolar disorder	500 (0.8)	622 (1.0)	581 (1.1)	676 (1.3)
Schizophrenia	23 (<0.1)	38 (0.1)	37 (0.1)	46 (0.1)
Substance use disorder	886 (1.5)	1314 (2.1)	1768 (3.3)	3388 (6.4)

Abbreviations: ACA, Patient Protection and Affordable Care Act; EPO, exclusive provider organization; HMO, health maintenance organization; MHPAEA, Mental Health Parity and Addiction Equity Act; POS, point of service; PPO, preferred provider organization.

**Figure 1.**
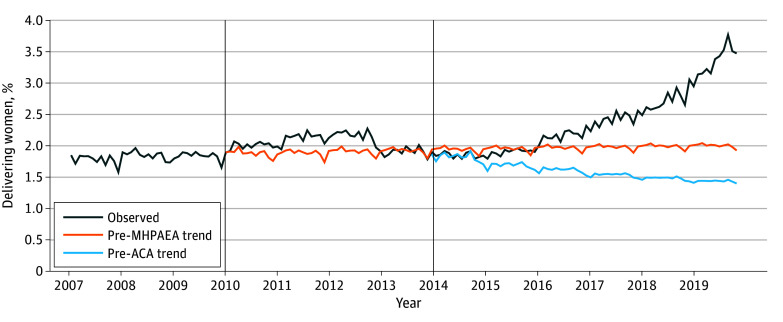
Percentage of All Privately Insured Women With Evidence of Any Psychotherapy Visits, by Month, 2007-2019 Observed indicates observed outcome trend accounting for the Mental Health Parity and Addiction Equity Act (MHPAEA) and Patient Protection and Affordable Care Act (ACA). Pre-MHPAEA trend indicates projected outcome trend stemming from the pre-MHPAEA period as if neither the MHPAEA nor ACA took place. Pre-ACA trend indicates projected outcome trend stemming from the pre-ACA trend as if the MHPAEA had occurred but the ACA did not take place. The vertical line at 2010 represents MHPAEA implementation; the vertical line at 2014 represents ACA implementation. Models were adjusted for autoregressive, seasonal autoregressive, moving average, and seasonal moving average.

Table 2 and Figure 1 show ARIMA models estimating any psychotherapy and per-visit OOPC for psychotherapy overall and among those with PMADs. We found a nonsignificant step change in the delivering women who received psychotherapy after MHPAEA implementation of 0.09% (95% CI, −0.04% to 0.21%; *P* = .16) and a nonsignificant slope change of delivering individuals who received psychotherapy of 0.00% per month (95% CI, −0.02% to 0.01%; *P* = .69) in the overall cohort. We found a nonsignificant step change in delivering women who received psychotherapy after ACA implementation of 0.11% (95% CI, −0.01% to 0.22%; *P* = .07) and a significantly increased slope change of delivering women who received psychotherapy of 0.03% per month (95% CI, 0.00%-0.05%; *P* = .02).

**Table 2.** Interrupted Time Series ARIMA Models Projecting Any Psychotherapy Visit and Patient OOPCs After MHPAEA and ACA Implementation Among Delivering Women Overall and With PMAD, 2007-2019^a^

Policy	Step change		Slope change		AIC	MAPE, %	Ljung-Box test	*df*	*P* value for overall model fit
	Estimate (95% CI)	*P* value	Estimate (95% CI)	*P* value					
**Any psychotherapy, %**									
Overall population									
MHPAEA	0.09 (−0.04 to 0.21)	.16	0.00 (−0.02 to 0.01)	.69	−378.04	2.61	10.01	12	.61
ACA	0.11 (−0.01 to 0.22)	.07	0.03 (0.00 to 0.05)	.02					
Women with PMADs									
MHPAEA	0.72 (0.26 to 1.18)	<.001	−0.05 (−0.09 to −0.02)	.01	79.87	2.54	9.74	12	.63
ACA	0.77 (0.26 to 1.27)	<.001	0.07 (0.02 to 0.12)	<.001					
**OOPCs for a psychotherapy visit, $**									
Overall population									
MHPAEA	1.95 (−0.20 to 4.11)	.08	−0.15 (−0.24 to −0.07)	<.001	615.52	3.11	4.85	12	.96
ACA	3.14 (1.56 to 4.73)	<.001	0.07 (0.02 to 0.12)	.01					
Women with PMADs									
MHPAEA	2.13 (−0.22 to 4.49)	.08	−0.22 (−0.32 to −0.12)	<.001	666.96	3.59	6.36	12	.90
ACA	2.54 (0.54 to 4.54)	<.001	0.10 (0.03 to 0.17)	<.001					

Abbreviations: ACA, Patient Protection and Affordable Care Act; AIC, Akaike Information Criteria; ARIMA, autoregressive integrated moving average; MAPE, mean absolute percent error; MHPAEA, Mental Health Parity and Addiction Equity Act; OOPC, out-of-pocket cost; PMAD, perinatal mood and anxiety disorders.

^a^ Models were adjusted for intercept, autoregressive, seasonal autoregressive, moving average, and seasonal moving average. AIC was used to assess model goodness of fit and parsimony, with the lowest AIC representing the best model fit.^42^ MAPE was used to measure model precision, with 5% as an indication that the forecast has acceptable accuracy, with lower scores representing better precision.^43^ Finally, Ljung-Box tests were conducted to assess whether autocorrelation remained, in which *P* > .05 fails to reject the null hypothesis that significant autocorrelation does not exist.^39^

**Figure 2.**
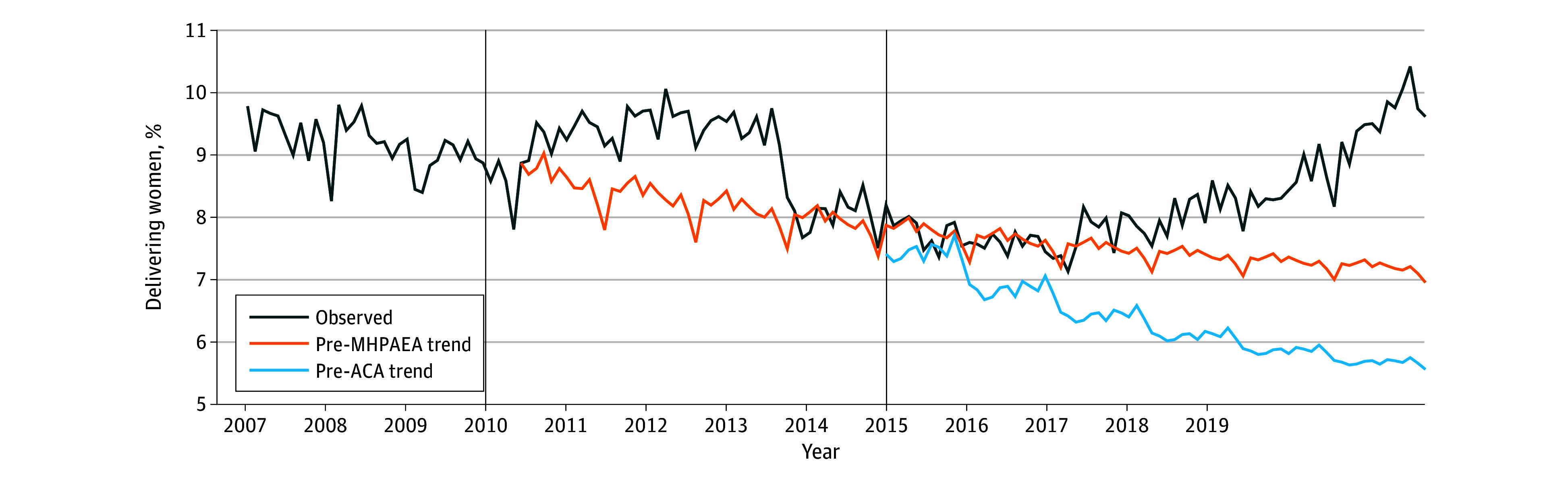
Percentage of Privately Insured Women With Perinatal Mood and Anxiety Disorders With Evidence of Any Psychotherapy Visits, by Month, 2007-2019 Observed indicates observed outcome trend accounting for the Mental Health Parity and Addiction Equity Act (MHPAEA) and Patient Protection and Affordable Care Act (ACA). Pre-MHPAEA trend indicates projected outcome trend stemming from the pre-MHPAEA period as if neither the MHPAEA nor ACA took place. Pre-ACA trend indicates projected outcome trend stemming from the pre-ACA trend as if the MHPAEA had occurred but the ACA did not take place. The vertical line at 2010 represents MHPAEA implementation; the vertical line at 2014 represents ACA implementation. Models were adjusted for autoregressive, seasonal autoregressive, moving average, and seasonal moving average.

Among those with PMADs, we found a significant step increase in delivering women who received psychotherapy after MHPAEA implementation of 0.72% (95% CI, 0.26%-1.18%; *P* = .002) and significantly decreased slope change of delivering individuals who received psychotherapy of −0.05% per month (95% CI, −0.09% to −0.02%; *P* = .01) (Figure 2). We found a significant step increase in delivering women who received psychotherapy after ACA implementation of 0.77% (95% CI, 0.26%-1.27%; *P* = .003) and a significantly increased slope change of delivering women who received psychotherapy of 0.07% per month (95% CI, 0.02%-0.12%; *P* = .005).

**Figure 3.**
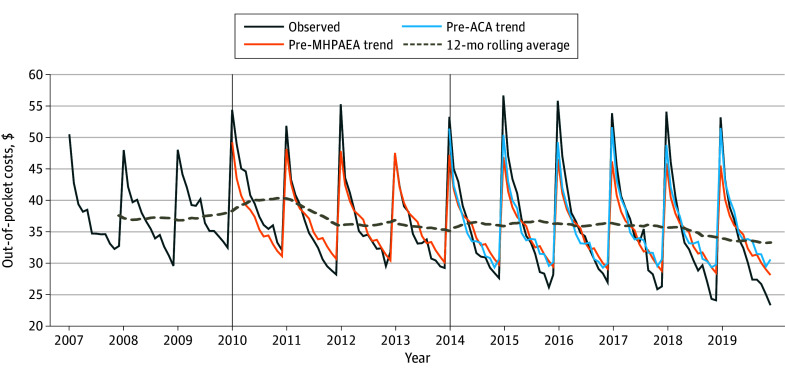
Per-Visit Out-of-Pocket Costs for Psychotherapy Among Privately Insured Women, by Month, 2007-2019 Observed indicates observed outcome trend accounting for the Mental Health Parity and Addiction Equity Act (MHPAEA) and Patient Protection and Affordable Care Act (ACA). Pre-MHPAEA trend indicates projected outcome trend stemming from the pre-MHPAEA period as if neither the MHPAEA nor ACA took place. Pre-ACA trend indicates projected outcome trend stemming from the pre-ACA trend as if the MHPAEA had occurred but the ACA did not take place. The vertical line at 2010 represents MHPAEA implementation; the vertical line at 2014 represents ACA implementation. Models were adjusted for autoregressive, seasonal autoregressive, moving average, and seasonal moving average. Costs standardized to 2019 dollars.

We found a nonsignificant step change in OOPCs per psychotherapy visit after MHPAEA implementation of $1.95 (95% CI, −$0.20 to $4.11; *P* = .08) and a significantly decreased slope change of OOPCs per psychotherapy visit of −$0.15 per month (95% CI, −$0.24 to −0.07; *P* < .001) in the overall cohort (Figure 3). We found a significant step increase in OOPCs per psychotherapy visit after ACA implementation of $3.14 (95% CI, $1.56 to $4.73; *P* < .001) and a significantly increased slope of OOPCs per psychotherapy visit of $0.07 per month (95% CI, $0.02-$0.12; *P* = .01).

Among those with PMADs, we found a nonsignificant step change in OOPCs per psychotherapy visit after MHPAEA implementation of $2.13 (95% CI, −$0.22 to $4.49; *P* = .08) and a significantly decreased slope change of OOPCs per psychotherapy visit of −$0.22 per month (95% CI, −$0.32 to −$0.12; *P* < .001) (eFigure 2 in Supplement 1). We found a significant step increase in OOPCs per psychotherapy visit after ACA implementation of $2.54 per month (95% CI, $0.54-$4.54; *P* = .01) and a significantly increased slope change in OOPCs per psychotherapy visit of $0.10 per month (95% CI, $0.03-$0.17; *P* = .004).

For overall context, we provide additional information on secondary outcomes in eFigures 3, 4, and 5 in Supplement 1. We illustrate increases in the total number of psychotherapy visits experienced monthly among privately insured delivering women (eFigure 3 in Supplement 1). We display the decreasing total number of psychotherapy visits per patient per month among patients who have at least 1 visit (eFigure 4 in Supplement 1). We illustrate decreasing per-visit standard cost (insurer covered) of psychotherapy visits per month (eFigure 5 in Supplement 1).

We dissected OOPCs into components relative to standard costs (eFigure 6 in Supplement 1) and discovered that most OOPCs seasonality stemmed from deductibles, which are typically higher at the beginning of a calendar year and decrease toward the year’s end. Although standard cost and overall OOPCs of psychotherapy remain relatively stable over time, the deductible component of OOPCs increased, whereas the copayment and coinsurance components slightly decreased.

Furthermore, we examined the ratio between OOPCs and standard costs over time (eFigure 7 in Supplement 1), which revealed that although absolute OOPCs for a psychotherapy visit remained relatively stable, OOPCs for all types of visits increased over time. Consequently, the ratio of OOPCs for a psychotherapy visit relative to OOPCs for all types of visits decreased.

## Discussion

Implementation of the MHPAEA and ACA was associated with changing access to and OOPCs for psychotherapy among delivering women; however, results varied by policy and between the 2 cohorts (eg, overall delivering population vs those with PMADs). We did not find a statistically significant immediate change associated with the MHPAEA or ACA in the overall delivering population, except for a steady increase in delivering women who received any psychotherapy after ACA. Among those with PMADs, a substantial association was found with the MHPAEA and ACA in those who received any psychotherapy. Psychotherapy rates immediately decreased after MHPAEA implementation then followed a sustained increase over time, whereas immediate and sustained increases occurred after ACA implementation.

We found similar results for the overall cohort and those with PMADs regarding OOPCs for psychotherapy visits. Both had no immediate change associated with MHPAEA implementation but had a sustained decrease after MHPAEA implementation in OOPCs over time; ACA implementation was associated with immediate and sustained increases in OOPCs.

Although the policies increased access to any psychotherapy, the greater number of people receiving visits coincided with fewer visits per person. One hypothesis suggests that the number of available MH clinicians may not have increased enough to meet the new demand^46^; future research should better characterize this trend. The lower standard cost may have further dampened any incentive to increase the number of clinicians. These forces may explain why those with PMADs may have experienced an immediate decrease in the proportion of those who received any psychotherapy immediately after MHPAEA implementation. Implementation of the ACA increased coverage for services, including psychotherapy, but did not prevent plans from increasing costs to patients, provided those increases did not disproportionately occur among MH services relative to medical and surgical care. Such costs could include those for the individual mandate and increased use of high-deductible health plans. It is also possible that changes in OOPCs for psychotherapy, including an increased proportion of those with no OOPCs, could have improved accessibility. However, we found that the ratio of OOPCs for psychotherapy relative to overall OOPCs decreased over time.

One should also review these findings in the context of substantial population MH burdens. Due to the MH care professional shortage, less than 30% of MH care needs are met.^47^ Approximately three-quarters of employers provide only 1 option for health insurance coverage, which limits employee choice.^23^ Furthermore, rampant health plan violations of the MHPAEA have existed for years and may have dampened the intended impact on access to MH services.^48,49^

### Limitations

This study has some limitations. The study did not address treatment adequacy or other forms of MH treatment, such as antidepressant medications, or optimal numbers of psychotherapy visits. We did not exclude individuals with other MH conditions from the study; however, this would likely bias our findings toward the null. Because we required individuals in study cohorts to have continuous enrollment for a 2-year period to measure treatment access throughout pregnancy and postpartum, our findings may not represent individuals with gaps in insurance coverage.

We could not assess plan-level MH or other benefits directly (ie, potential change in use of high-deductible health plans) and therefore included plans with evidence of provision of MH services to any individual. We could not account for severity of PMADs and used diagnoses rather than screening instruments designed to assess depression and anxiety diagnoses. If our data missed individuals with true underlying PMADs that remained undiagnosed, our findings would be biased toward the null. We note that although the *ICD-9-CM* to *ICD-10-CM* switch occurred in 2015, shortly after ACA implementation, this change did not affect PMAD diagnosis or psychotherapy codes; however, it may have influenced the proportion of individuals diagnosed with comorbid illnesses, which might explain the large increase in comorbidities at the end of the study period (Table 1; eTable 2 in Supplement 1). Finally, our analyses focused on privately insured individuals and therefore may not represent findings among individuals with public insurance coverage, such as Medicaid.

## Conclusions

In this cross-sectional, observational study of commercially insured delivering women in the US, use of psychotherapy increased during the study period overall and among individuals with PMAD diagnoses. For psychotherapy, OOPCs increased at implementation of both policies, with decreases after MHPAEA but not ACA implementation. Implementation of the ACA likely had a larger effect than MHPAEA implementation, which was to be expected given how the ACA extended the impact of the MHPAEA and closed its loopholes. Given ongoing challenges associated with appropriate MH care for delivering women with PMADs, policymakers, health plans, and clinicians should continue efforts to improve access to evidence-based treatment for PMADs and continue to seek health system and policy avenues to mitigate the excess burden of PMADs in this population.

## Notes

Supplement 1. **eTable 1.** Codes Used to Create Delivery Cohort, PMAD Diagnoses, Psychotherapy Visits, and Bateman Comorbidity Index **eTable 2.** Unadjusted Sociodemographic and Clinical Characteristics Associated With Deliveries Among Privately Insured Delivering Individuals With and Without PMAD, 2007-2019 **eFigure 1.** Study Numerator, Denominator, and Monthly Cohorts **eFigure 2.** Per Visit OOPC for Psychotherapy Among Privately Insured Women With PMAD, by Month, 2007-2019 **eFigure 3.** Total Psychotherapy Visits Among Delivering Privately Insured Women, by Month, 2007-2019 **eFigure 4.** Total Psychotherapy Visits per Patient per Month for Patients With One or More Visits Among Delivering Privately Insured Women, by Month, 2007-2019 **eFigure 5.** Per Visit Standard Cost of Psychotherapy Visits per Month Among Delivering Privately Insured Women, 2007-2019 (Inflation Adjusted to 2019 $) **eFigure 6.** Per Visit Out-of-Pocket Costs and Standard Costs of Psychotherapy Visits per Month Among Delivering Privately Insured Women, 2007-2019 (Inflation Adjusted to 2019 $) **eFigure 7.** Per Visit Ratio Between Out-of-Pocket-Costs and Standard Costs of Psychotherapy Visits per Month Among Delivering Privately Insured Women, 2007-2019 (Inflation Adjusted to 2019 $)

Supplement 2.
Data Sharing Statement
