# Supplementary material for: Perinatal Psychotherapy Use and Costs Before and After Federally Mandated Health Insurance Coverage
Source: JAMA Netw Open. 2024 Aug 9;7(8):e2426802. doi: 10.1001/jamanetworkopen.2024.26802 (PMC11316231; doi:10.1001/jamanetworkopen.2024.26802)
Supplement: Supplement 1. — eTable 1. Codes Used to Create Delivery Cohort, PMAD Diagnoses, Psychotherapy Visits, and Bateman Comorbidity Index eTable 2. Unadjusted Sociodemographic and Clinical Characteristics Associated With Deliveries Among Privately Insured Delivering Individuals With and Without PMAD, 2007-2019 eFigure 1. Study Numerator, Denominator, and Monthly Cohorts eFigure 2. Per Visit OOPC for Psychotherapy Among Privately Insured Women With PMAD, by Month, 2007-2019 eFigure 3. Total Psychotherapy Visits Among Delivering Privately Insured Women, by Month, 2007-2019 eFigure 4. Total Psychotherapy Visits per Patient per Month for Patients With One or More Visits Among Delivering Privately Insured Women, by Month, 2007-2019 eFigure 5. Per Visit Standard Cost of Psychotherapy Visits per Month Among Delivering Privately Insured Women, 2007-2019 (Inflation Adjusted to 2019 $) eFigure 6. Per Visit Out-of-Pocket Costs and Standard Costs of Psychotherapy Visits per Month Among Delivering Privately Insured Women, 2007-2019 (Inflation Adjusted to 2019 $) eFigure 7. Per Visit Ratio Between Out-of-Pocket-Costs and Standard Costs of Psychotherapy Visits per Month Among Delivering Privately Insured Women, 2007-2019 (Inflation Adjusted to 2019 $) [file jamanetwopen-e2426802-s001.pdf]

Supplementary Online Content

Zivin K, Zhang X, Tilea A, et al. Perinatal psychotherapy use and costs before and after federally mandated health insurance coverage. *JAMA Netw Open*. 2024;7(8):e2426802. doi:10.1001/jamanetworkopen.2024.26802

eTable 1. Codes Used to Create Delivery Cohort, PMAD Diagnoses, Psychotherapy Visits, and Bateman Comorbidity Index
eTable 2. Unadjusted Sociodemographic and Clinical Characteristics Associated With Deliveries Among Privately Insured Delivering Individuals With and Without PMAD, 2007-2019[a]
eFigure 1. Study Numerator, Denominator, and Monthly Cohorts
eFigure 2. Per visit OOPC for Psychotherapy Among Privately Insured Women With PMAD, by Month, 2007-2019
eFigure 3. Total Psychotherapy Visits Among Delivering Privately Insured Women, by Month, 2007-2019
eFigure 4. Total Psychotherapy Visits per Patient per Month for Patients With One or More Visits Among Delivering Privately Insured Women, by Month, 2007-2019
eFigure 5. Per Visit Standard Cost of Psychotherapy Visits per Month Among Delivering Privately Insured Women, 2007-2019 (Inflation Adjusted to 2019 $)
eFigure 6. Per Visit Out-Of-Pocket Costs and Standard Costs of Psychotherapy Visits per Month Among Delivering Privately Insured Women, 2007-2019 (Inflation Adjusted to 2019 $)
eFigure 7. Per Visit Ratio Between Out-of-Pocket-Costs and Standard Costs of Psychotherapy Visits per Month Among Delivering Privately Insured Women, 2007-2019 (Inflation Adjusted to 2019 $)

This supplementary material has been provided by the authors to give readers additional information about their work.

© 2024 Zivin K et al. *JAMA Network Open.*

eTable 1. Codes Used to Create Delivery Cohort, PMAD Diagnoses, Psychotherapy Visits, and Bateman Comorbidity Index

Deliveries

Included: Cesarean and vaginal delivery

	Cesarean delivery	Vaginal delivery
ICD-9-CM procedures	740, 741, 742, 744, 7499	
ICD-10-CM procedures	10D00Z0, 10D00Z1, 10D00Z2	10D07Z3, 10D07Z4, 10D07Z5, 10D07Z6, 10D07Z7, 10D07Z8, 10E0ZZ
DRG	370, 371, 765, 766, 540, 5401, 5402, 5403, 5404	372, 373, 374, 375, 767 768, 774, 775, 541, 542, 560, 5411, 5412, 5413, 5414, 5421, 5422, 5423, 5424, 5601, 5602, 5603
CPT	59510, 59514, 59515, 59618, 59620, 59622	59400, 59409, 59410, 59610, 59612, 59614

Included: Pre-term birth

ICD 10 diagnoses	O601, O6010, O60100, O60101, O60102, O60103, O60104, O60105, O60109, O6012, O60120, O60121, O60122, O60123, O60124, O60125, O60129, O6013, O60130, O60131, O60132, O60133, O60134, O60135, O60139, O6014, O60140, O60141, O60142, O60143, O60144, O60145, O60149
ICD 9 diagnoses	64420, 64421

Excluded: abortion, dilatation and curettage, and stillbirth

ICD procedures and CPT codes	Removal of ectopic pregnancy	59120, 59121, 59130, 59135, 59136, 59140, 59150, 59151, 743
	Abortion (termination of pregnancy)	59840, 59841, 59850, 59851, 59852, 59855, 59856, 59857, 10A00ZZ, 10A03ZZ, 10A04ZZ, 10A07Z, 10A07ZZ, 10A07ZZ, 10A08ZZ, 10A08ZZ, 6901, 6951, 7491, 750
	Dilatation and curettage (D&C); aspiration after delivery or abortion	59812, 59820, 59821, 59830
ICD 9 and 10 diagnoses	Removal of ectopic pregnancy	O0000, O0001, O00101, O00102, O00109, O00111, O00112, O00119, O00201, O00202, O00209, O00211, O00212, O00219, O0080, O0081, O0090, O0091, 63300, 63301, 63310, 63311, 63320, 63321, 63380, 63381, 63390, 63391,
	Abortion (termination of pregnancy)	O030, O031, O032, O0330, O0331, O0332, O0333, O0334, O0337, O0339, O034, O035, O036, O037, O0380, O0381, O0382, O0383, O0384, O0385, O0386, O0387, O0388, O0389, O039, 63400, 63401, 63402, 63410, 63411, 63412, 63420, 63421, 63422, 63430, 63431, 63432, 63440, 63441, 63442, 63450, 63451, 63452, 63460, 63461, 63462, 63470, 63471, 63472, 63480, 63481, 63482, 63490, 63491, 63492, O045, O045, O045, O046, O046, O046, O047, O047, O047, O0480, O0480, O0480, O0481, O0481, O0481, O0482, O0482, O0482, O0483, O0483, O0483, O0484, O0484, O0484, O0485, O0486, O0487, O0488, O0489, O0489, O0489, O070, O071, O072, O0730, O0731, O0732, O0733, O0734, O0735, O0736, O0737, O0738, O0739, O074, Z332, Z332, Z332, 63500, 63501, 63502, 63510, 63511, 63512, 63520, 63521, 63522, 63530, 63531, 63532, 63540, 63541, 63542, 63550, 63551, 63552, 63560, 63561, 63562, 63570, 63571, 63572, 63580, 63581, 63582, 63590, 63591, 63592, 63600, 63601, 63602, 63610, 63611, 63612, 63620, 63621, 63622, 63630, 63631, 63632, 63640, 63641, 63642, 63650, 63651, 63652, 63660, 63661, 63662, 63670, 63671, 63672, 63680, 63681, 63682, 63690, 63691, 63692, 63700, 63701, 63702, 63710, 63711, 63712, 63720, 63721, 63722, 63730, 63731, 63732, 63740, 63741, 63742, 63750, 63751, 63752, 63760, 63761, 63762, 63770, 63771, 63772, 63780, 63781, 63782, 63790, 63791, 63792, 6380, 6381, 6382, 6383, 6384, 6385, 6386, 6387, 6388, 6389, O080, O081, O082, O083, O084, O085, O086, O087, O0881, O0882, O0883, O0889, O089, 6390, 6391, 6392, 6393, 6394, 6395, 6396, 6398, 6399,
	Other complications	O019, O021, O0289, Z371, Z374, Z377, 630, 6310, 6318, 632, V271, V274, V277, A34

© 2024 Zivin K et al. *JAMA Network Open.*

PMAD

Group	ICD Diagnosis Code
Anxiety	300, 308, 313, 293, F06, F40, F41, F42, F43, F48, R45
Depression	311, 296, 300, F32, F33

Psychotherapy visits

**CPT/HCPCS**	90804, 90805, 90806, 90807, 90808, 90809, 90810, 90811, 90812, 90813, 90814, 90815, 90816, 90817, 90818, 90819, 90820, 90821, 90822, 90823, 90824, 90826, 90827, 90828, 90829, 90832, 90833, 90834, 90836, 90837, 90838, 90839, 90840, 90841, 90842, 90843, 90844, 90846, 90847, 90849, 90853, 90855

Bateman comorbidity

ICD-9 diagnosis codes	291, 303, 3050, 493, 394, 395, 396, 397, 424, 42822, 42823, 42832, 42833, 42842, 42843, 412, 413, 414, 581, 582, 583, 585, 587, 588, 6462, 7450, 7451, 7452, 7453, 7454, 7455, 7456, 7457, 7458, 7459, 7460, 7461, 7462, 7463, 7464, 7465, 7466, 7467, 7468, 7469, 7470, 7471, 7472, 7473, 7474, 6485, 304, 3052, 3053, 3054, 3055, 3056, 3057, 3058, 3059, 6483, 6423, 042, V08, 6424, 6427, 651, V272, V273, V274, V275, V276, V277, V278, 6410, 6411, 250, 6480, 401, 402, 403, 404, 405, 6420, 6421, 6422, 6427, 6542, 4160, 4168, 4169, 6425, 6466, 2824, 2826, 7100
ICD-10 diagnosis codes	F10, J44, J45, I05, I06, I07, I08, I09, I34, I35, I36, I37, I38, I39, I500, I20, I25, N022, N03, N04, N05, N08, N171, N172, N18, N25, O268, Q20, Q21, Q22, Q23, Q24, Q25, Q26, O994, F11, F12, F13, F14, F15, F16, F18, F19, O13, O16, B20, B24, O987, Z21, O11, O14, O30, O31, Z372, Z373, Z374, Z375, Z376, Z377, Z3790, O44, E10, E11, O245, O246, O247, I10, I11, I12, I13, I15, O10, O11, O3420, I270, I272, I278, I279, O14, O15, D56, D57, M32

References[1-12]

eTable 2. Unadjusted Sociodemographic and Clinical Characteristics Associated With Deliveries Among Privately Insured Delivering Individuals With and Without PMAD, 2007-2019[a]

	2007		2019	
	**PMAD (n = 8,585)**	**No PMAD (n = 51,499)**	**PMAD (n = 13,257)**	**No PMAD (n = 39,292)**
	**No. (%)**	**No. (%)**	**No. (%)**	**No. (%)**
**Age**				
15-24	904 (10.55)	4,921(9.56)	1587 (11.97)	3,462 (8.81)
25-39	7,162 (83.55)	43,832 (85.18)	10,847 (81.83)	33,522 (85.33)
40-44	506 (5.90)	2,707 (5.26)	822 (6.20)	2,302 (5.86)
**Race/Ethnicity**				
Asian	208 (2.42)	3,716 (7.22)	428 (3.23)	3,153 (8.02)
Black	559 (6.51)	4,417 (8.58)	888 (6.70)	3,094 (7.87)
Hispanic	812 (9.46)	6,311 (12.25)	1,344 (10.14)	5,253 (13.37)
White	5,941 (69.20)	30,319 (58.87)	8,795 (66.34)	21,826 (55.55)
Unknown	1,065 (12.41)	6,736 (13.08)	1,802 (13.59)	5,966 (15.18)
**Region**				
Midwest	2,488 (28.98)	12,343 (23.97)	4,107 (30.98)	10,455 (26.61)
Northeast	**	5,144 (9.99)	1,364 (10.29)	4,096 (10.42)
South	3,689 (42.97)	23,301 (45.25)	5,204 (39.25)	16,125 (41.04)
West	1,436 (16.73)	10,662 (20.70)	2,569 (19.38)	8,484 (21.59)
Unknown	**	49 (0.10)	13 (0.10)	132 (0.34)
**Insurance Plan Type**				
EPO	1,413 (16.46)	8,760 (17.01)	1,735 (13.09)	5,169 (13.16)
HMO	1,352 (15.75)	8,102 (15.73)	1,416 (10.68)	4,270 (10.87)
IND/OTH/PPO	455 (5.30)	2,371 (4.60)	438 (3.30)	926 (2.36)
POS	5,365 (62.49)	32,266 (62.65)	9,668 (72.93)	28,927 (73.62)
**Clinical characteristics**				
Mode of delivery: cesarean	3,470 (40.42)	18,243 (35.42)	4,859 (36.65)	12,995 (33.07)
Bateman score >0	1,895 (22.07)	7,220 (14.02)	5,005 (37.75)	10,390 (26.44)
Suicidality	110 (1.29)	**	352 (2.66)	**
Bipolar disorder	352 (4.12)	148 (0.29)	566 (4.27)	110 (0.28)
Schizophrenia	16 (0.19)	**	43 (0.32)	**
Substance use disorder	420 (4.89)	466 (0.90)	1,871 (14.11)	1,517 (3.86)

[a] We include data from 2006-2020 but require a year before and year after delivery of continuous enrollment, therefore, we present deliveries in 2007-2019

** We censored cell sizes <10 and the next smallest cell in that category.

© 2024 Zivin K et al. *JAMA Network Open.*

eFigure 1. Study Numerator, Denominator, and Monthly Cohorts

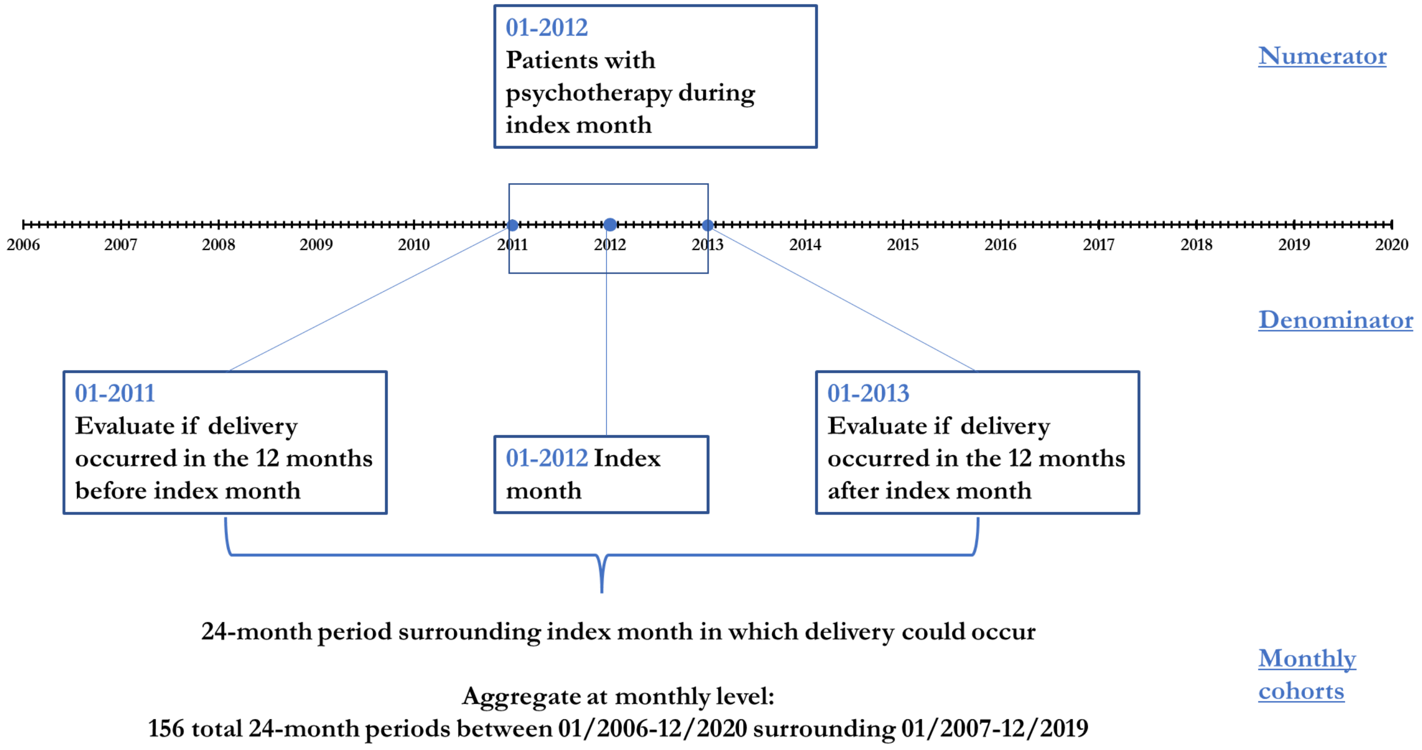

Notes

- Tick marks represent months

© 2024 Zivin K et al. *JAMA Network Open.*

eFigure 2. Per Visit OOPC for Psychotherapy Among Privately Insured Women With PMAD, by Month, 2007-2019

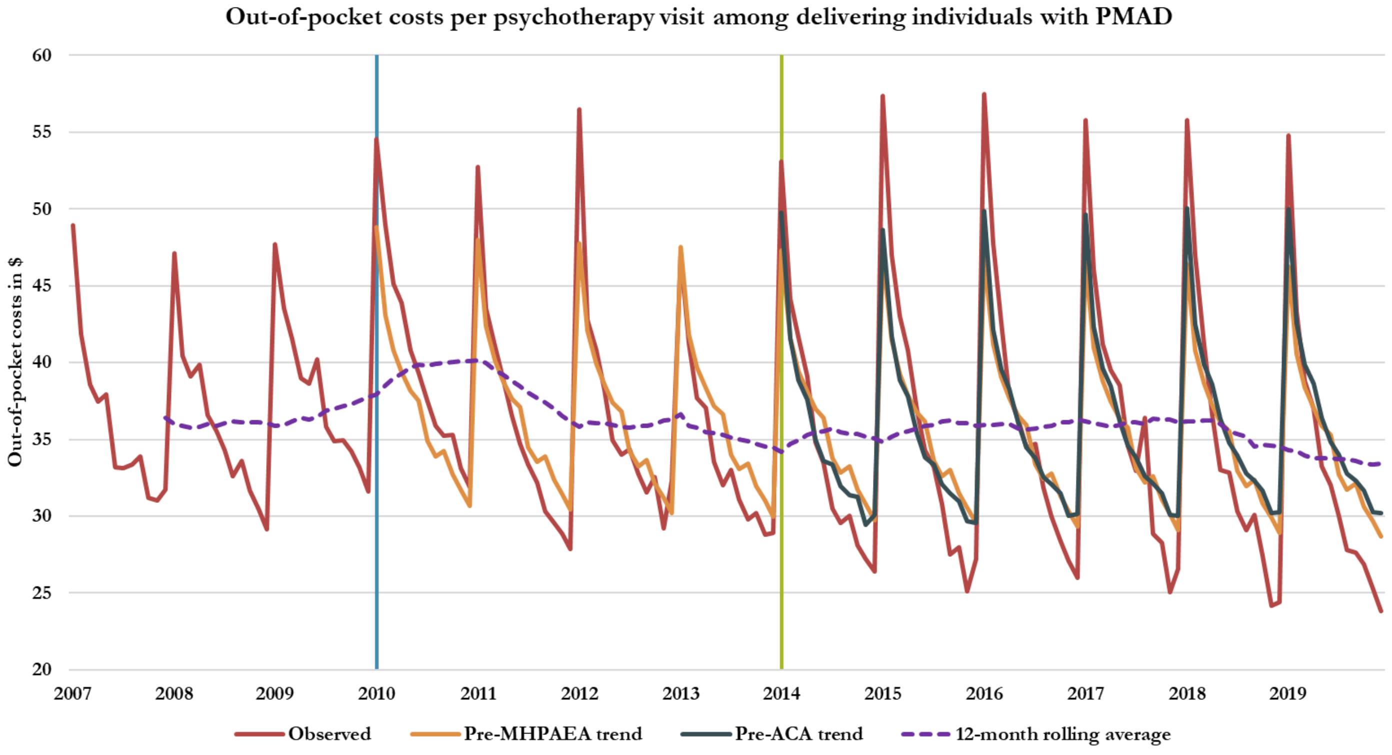

Notes

- Observed = observed outcome trend accounting for MHPAEA and ACA
- Pre-MHPAEA trend = predicted outcome trend stemming from the pre-MHPAEA period as if neither MHPAEA nor ACA took place
- Pre-ACA trend = predicted outcome trend stemming from the pre-ACA trend as if MHPAEA had occurred, but ACA did not take place
- Period 1=2007-2010; Period 2=2010-2014; Period 3=2014-2019
- 2010 vertical blue line represents MHPAEA implementation; 2014 vertical green line represents ACA implementation
- Models adjusted for Auto Regressive (AR), Seasonal Auto Regressive (SAR), Moving Average (MA), and Seasonal Moving Average (SMA)
- Costs standardized to 2019 dollars

© 2024 Zivin K et al. *JAMA Network Open.*

eFigure 3. Total Psychotherapy Visits Among Delivering Privately Insured Women, by Month, 2007-2019

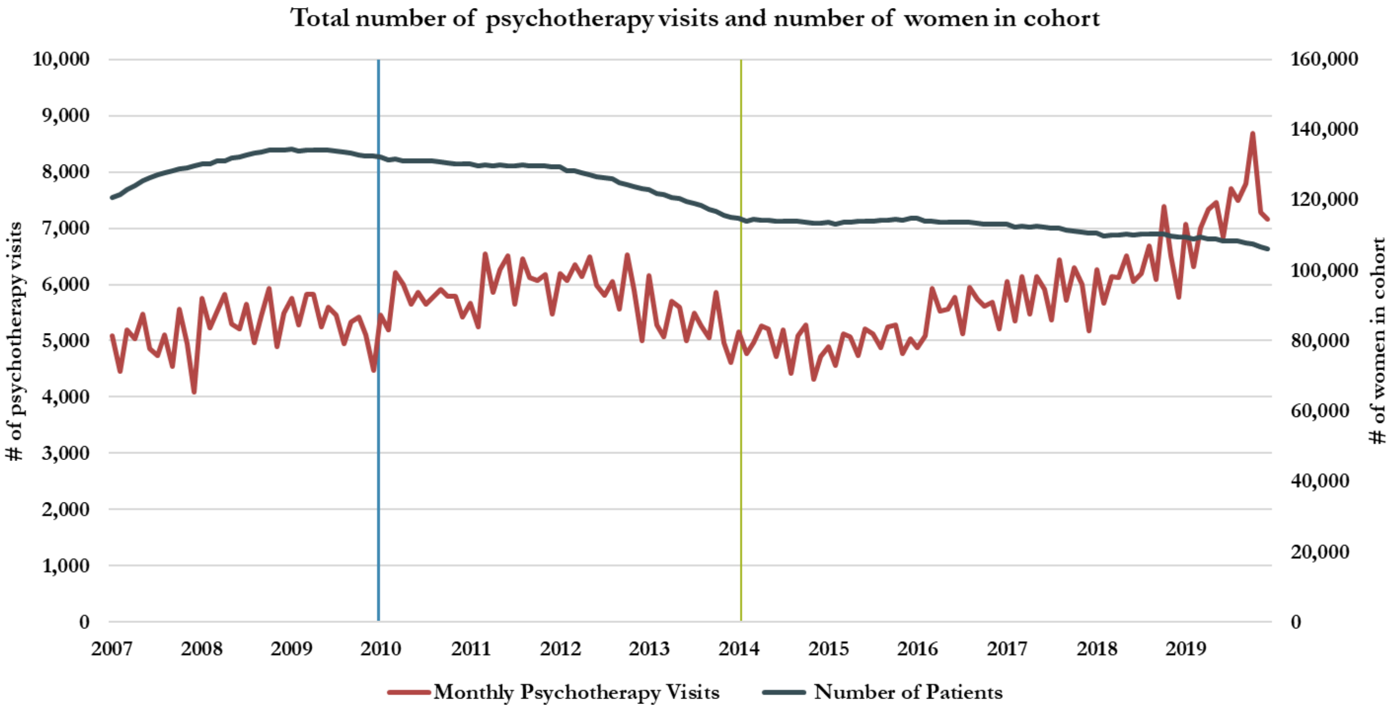

Notes

- Period 1=2007-2010; Period 2=2010-2014; Period 3=2014-2019
- 2010 vertical blue line represents MHPAEA implementation; 2014 vertical green line represents ACA implementation
- Models adjusted for Auto Regressive (AR), Seasonal Auto Regressive (SAR), Moving Average (MA), and Seasonal Moving Average (SMA)

© 2024 Zivin K et al. *JAMA Network Open.*

eFigure 4. Total Psychotherapy Visits per Patient per Month for Patients With One or More Visits Among Delivering Privately Insured Women, by Month, 2007-2019

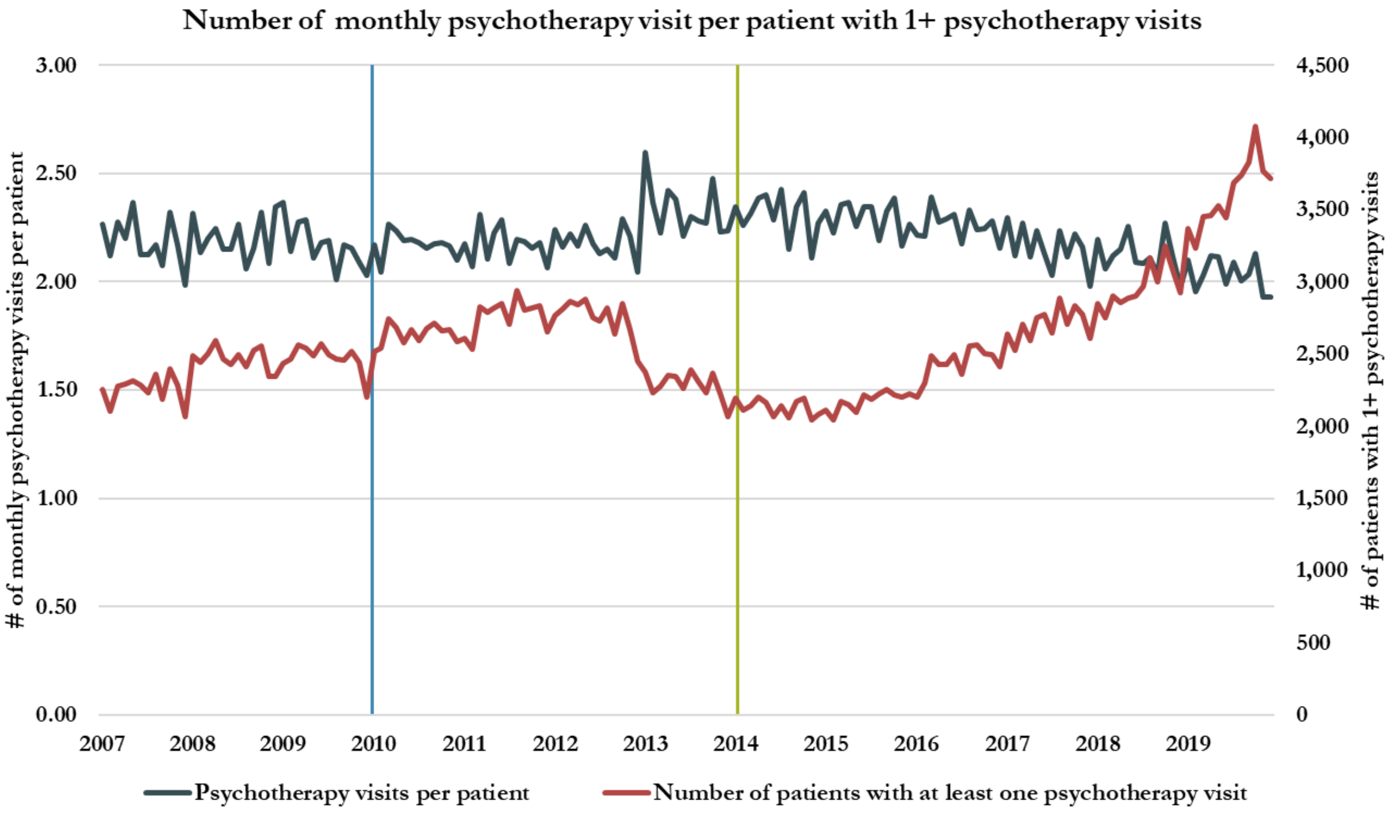

Notes

- Period 1=2007-2010; Period 2=2010-2014; Period 3=2014-2019
- 2010 vertical blue line represents MHPAEA implementation; 2014 vertical green line represents ACA implementation
- Models adjusted for Auto Regressive (AR), Seasonal Auto Regressive (SAR), Moving Average (MA), and Seasonal Moving Average (SMA)

© 2024 Zivin K et al. *JAMA Network Open.*

eFigure 5. Per Visit Standard Cost of Psychotherapy Visits per Month Among Delivering Privately Insured Women, 2007-2019 (Inflation Adjusted to 2019 $)

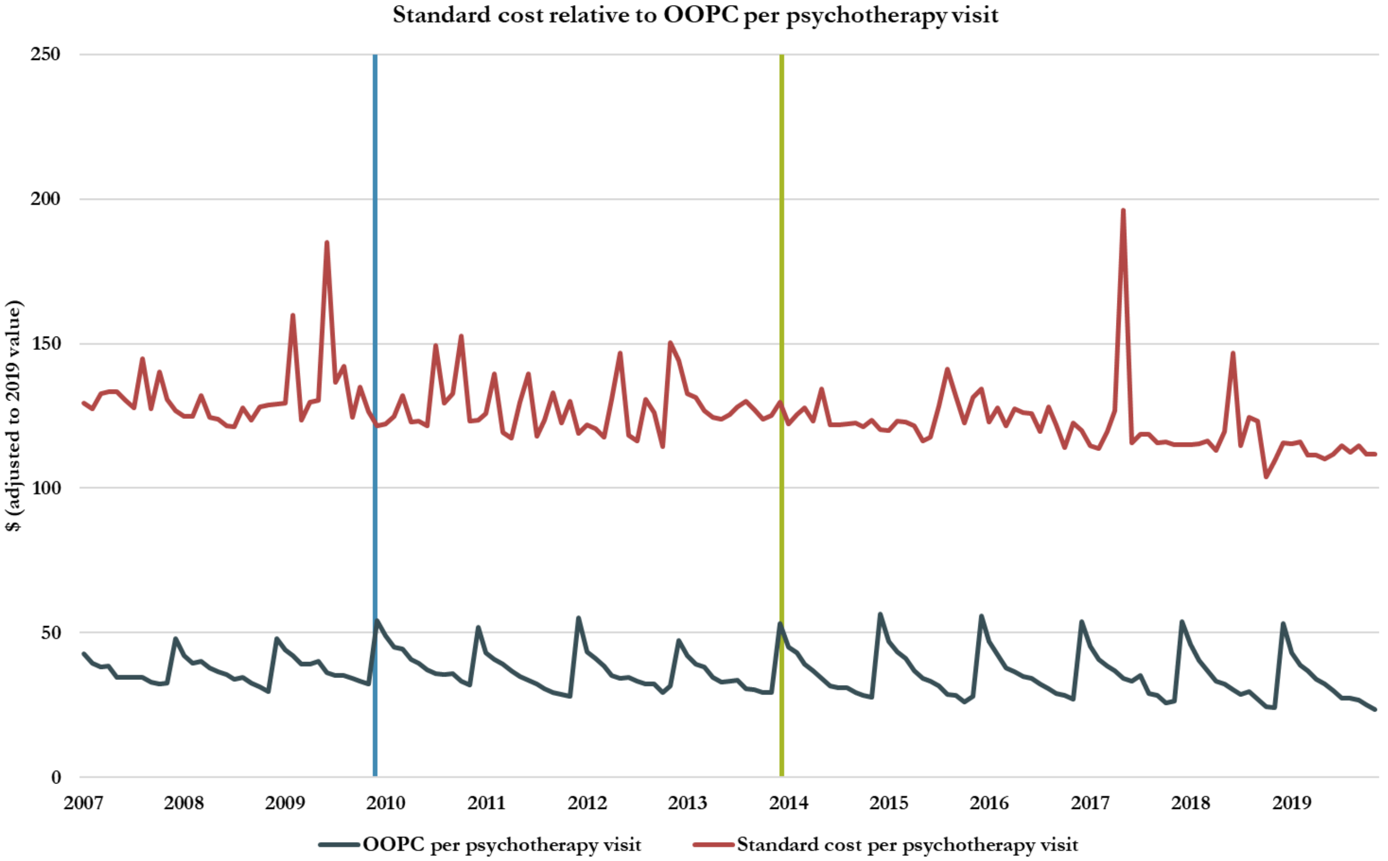

Notes

- Period 1=2007-2010; Period 2=2010-2014; Period 3=2014-2019
- 2010 vertical blue line represents MHPAEA implementation; 2014 vertical green line represents ACA implementation
- Models adjusted for Auto Regressive (AR), Seasonal Auto Regressive (SAR), Moving Average (MA), and Seasonal Moving Average (SMA)
- Costs standardized to 2019 dollars

© 2024 Zivin K et al. *JAMA Network Open.*

eFigure 6. Per Visit Out-Of-Pocket Costs and Standard Costs of Psychotherapy Visits per Month Among Delivering Privately Insured Women, 2007-2019 (Inflation Adjusted to 2019 $)

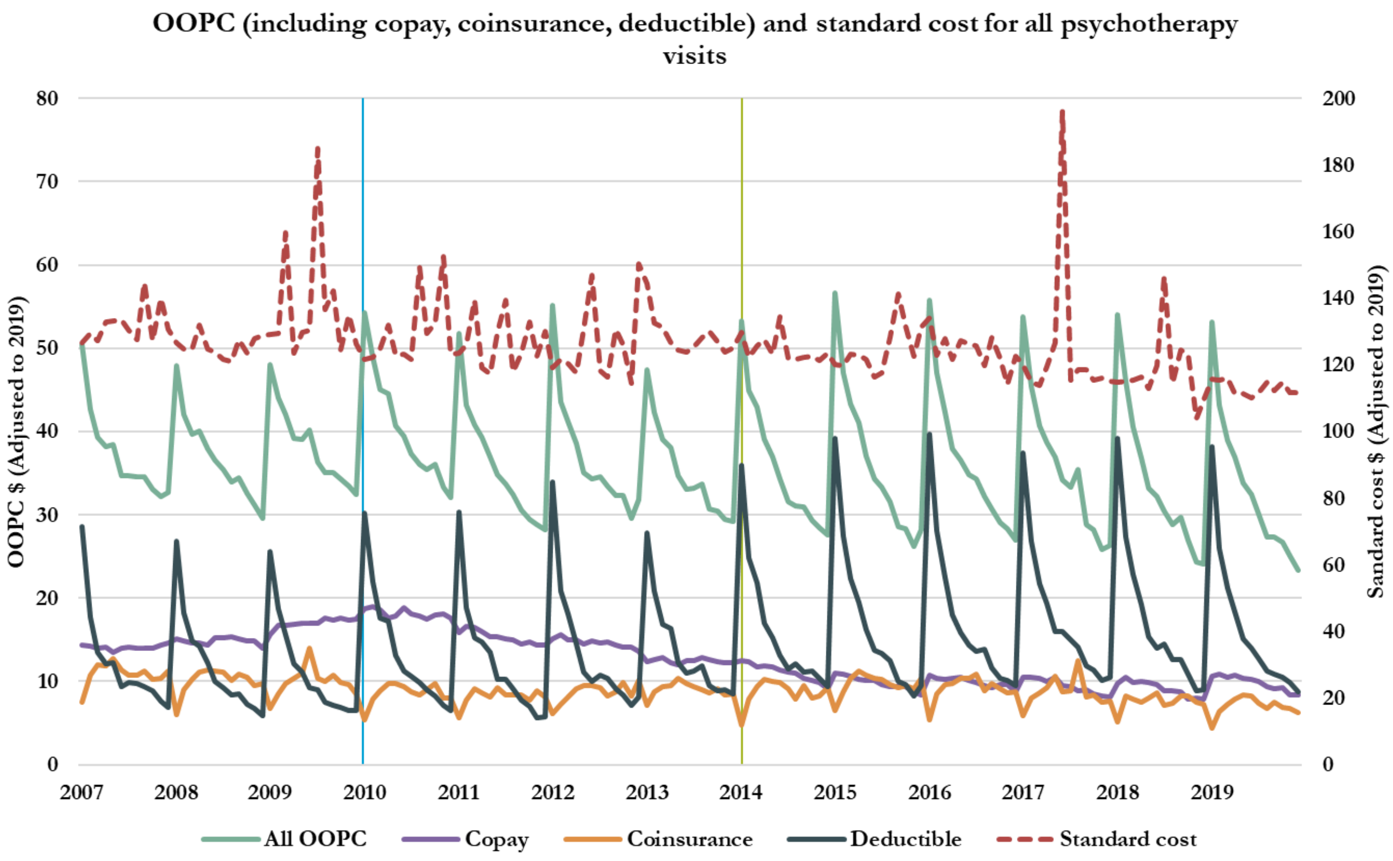

Notes

- Period 1=2007-2010; Period 2=2010-2014; Period 3=2014-2019
- 2010 vertical blue line represents MHPAEA implementation; 2014 vertical green line represents ACA implementation
- Models adjusted for Auto Regressive (AR), Seasonal Auto Regressive (SAR), Moving Average (MA), and Seasonal Moving Average (SMA)
- Costs standardized to 2019 dollars

© 2024 Zivin K et al. *JAMA Network Open.*

eFigure 7. Per Visit Ratio Between Out-of-Pocket-Costs and Standard Costs of Psychotherapy Visits per Month Among Delivering Privately Insured Women, 2007-2019 (Inflation Adjusted to 2019 $)

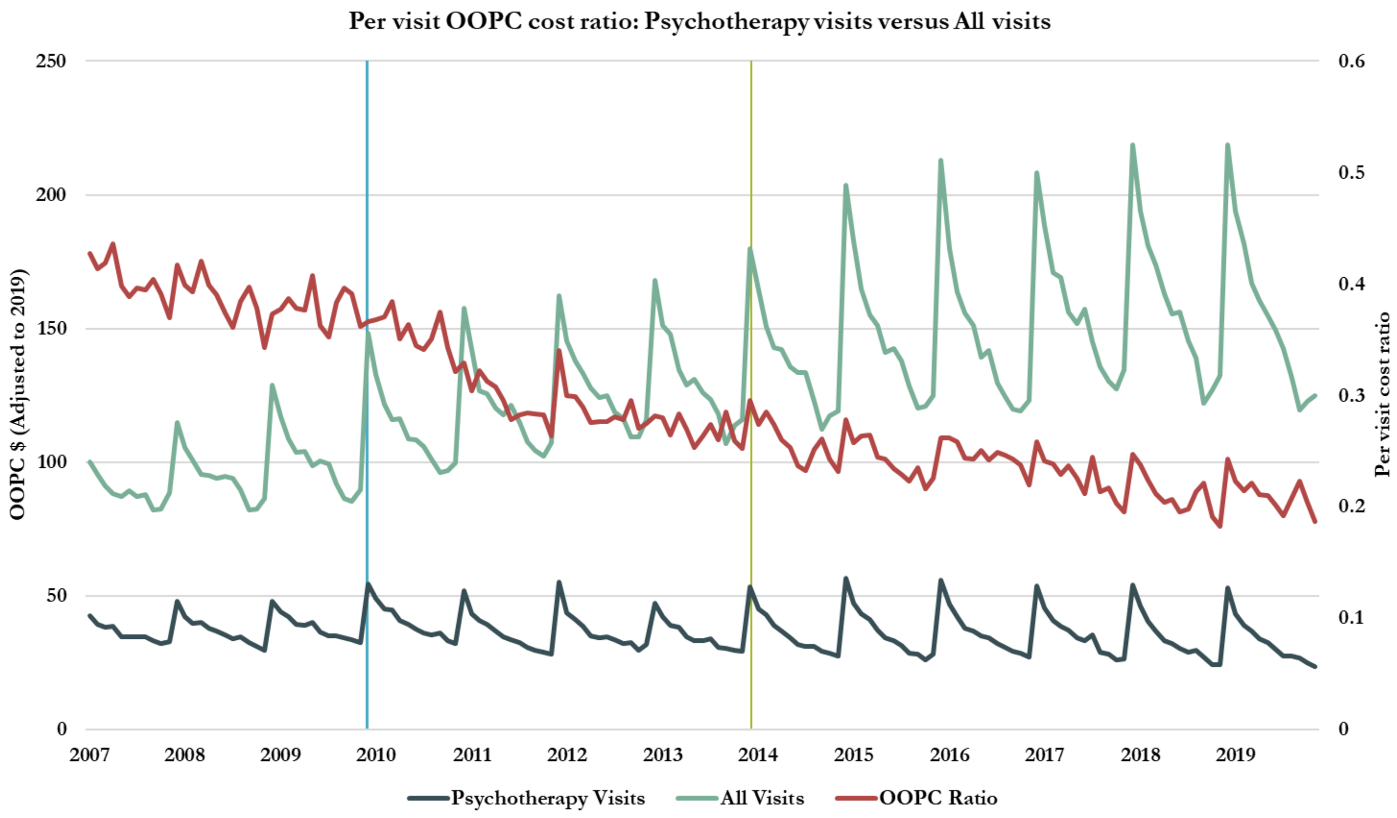

Notes

- Period 1=2007-2010; Period 2=2010-2014; Period 3=2014-2019
- 2010 vertical blue line represents MHPAEA implementation; 2014 vertical green line represents ACA implementation
- Models adjusted for Auto Regressive (AR), Seasonal Auto Regressive (SAR), Moving Average (MA), and Seasonal Moving Average (SMA)
- Costs standardized to 2019 dollars

© 2024 Zivin K et al. *JAMA Network Open.*

References

1. Agency for Healthcare Research and Quality. Beta Clinical Classifications Software (CCS) for ICD-10-CM/PCS. Healthcare Cost and Utilization Project (HCUP). www.hcup-us.ahrq.gov/toolssoftware/ccs10/ccs10.jsp

2. Olfson M, Kroenke K, Wang S, Blanco C. Trends in office-based mental health care provided by psychiatrists and primary care physicians. *Journal of Clinical Psychiatry*. 2014;75(3):247-53.

3. Centers for Medicare and Medicaid Services. 2018 ICD-10 CM and GEMs. 2018. https://www.cms.gov/Medicare/Coding/ICD10/2018-ICD-10-CM-and-GEMs

4. Xu X, Gariepy A, Lundsberg LS, et al. Wide Variation Found In Hospital Facility Costs For Maternity Stays Involving Low-Risk Childbirth. *Health Affairs*. 2015/07/01 2015;34(7):1212-1219. doi:10.1377/hlthaff.2014.1088

5. Healthcare Cost and Utilization Project. Clinical Classifications Software (CCS) for ICD-9-CM. https://www.hcup-us.ahrq.gov/toolssoftware/ccs/ccs.jsp

6. Canadian Institute for Health Information. Canadian Coding Standards for Version 2018 ICD-10-CA and CCI. A Guide to Obstetrical Coding. 2022.

7. Centers for Medicare and Medicaid Services. 2018 ICD-10 PCS and GEMs. 2018. https://www.cms.gov/Medicare/Coding/ICD10/2018-ICD-10-PCS-and-GEMs

8. Kukla M, Bond GR. A randomized controlled trial of evidence-based supported employment: Nonvocational outcomes. *Journal of Vocational Rehabilitation*. 2013;38(2):91-98.

9. Camelo Castillo W, Boggess K, Stürmer T, Brookhart MA, Benjamin DKJ, Jonsson Funk M. Trends in Glyburide Compared With Insulin Use for Gestational Diabetes Treatment in the United States, 2000–2011. *Obstetrics and Gynecology*. 2014;123(6):1177-1184.

10. Averill RF, McCullough EC, Goldfield N, et al. *3M™ APR DRG Classification System Version 31.0 Methodology Overview*. 2013. https://www.hcup-us.ahrq.gov/db/nation/nis/grp031_aprdrg_meth_ovrview.pdf

11. Bateman BT, Mhyre JM, Hernandez-Diaz S, et al. Development of a comorbidity index for use in obstetric patients. *Obstetrics and Gynecology*. 2013;122(5):957-65.

12. Joyce NR, Schuler MS, Hadland SE, Hatfield LA. Variation in the 12-Month Treatment Trajectories of Children and Adolescents After a Diagnosis of Depression. *JAMA Pediatrics*. 2018;172(1):49-56.

© 2024 Zivin K et al. *JAMA Network Open.*
