# Supplementary material for: Perinatal Psychotherapy Use and Costs Before and After Federally Mandated Health Insurance Coverage
Source: JAMA Netw Open. 2024 Aug 9;7(8):e2426802. doi: 10.1001/jamanetworkopen.2024.26802 (PMC11316231; doi:10.1001/jamanetworkopen.2024.26802)
Supplement: Supplement 2. — Data Sharing Statement [file jamanetwopen-e2426802-s002.pdf]

Data Sharing Statement

Data
Data available: No

Additional Information
Explanation for why data not available: The data that support the findings of this study are available from Optum's de-identified Clinformatics® Data Mart Database (CDM). Restrictions apply to the availability of these data, which were used under license for this study, and thus are not publicly available.
